# Profiling of Known and Novel microRNAs in an Oleaginous Crop Native to the Amazon Basin, Sacha Inchi (*Plukenetia volubilis*), Through smallRNA-Seq

**DOI:** 10.3390/genes16040417

**Published:** 2025-03-31

**Authors:** Richard Estrada, Lila Rodriguez, Yolanda Romero, Linda Arteaga, Domingo Ruelas-Calloapaza, Filiberto Oha-Humpiri, Nils Flores, Pedro Coila, Carlos I. Arbizu

**Affiliations:** 1 Dirección de Desarrollo Tecnológico Agrario, Instituto Nacional de Innovación Agraria (INIA), Lima 15024, Peru; limrp.bioinfo@gmail.com (L.R.); yolanda.bioinfo@gmail.com (Y.R.); linda.arteaga@bioinfo.net.pe (L.A.); 2 Instituto de Investigación en Bioinformática y Bioestadística (BIOINFO), Lima 15024, Peru; 3 Facultad de Medicina Veterinaria y Zootecnia, Universidad Nacional del Altiplano de Puno, Puno 21001, Peru; druela@unap.edu.pe (D.R.-C.); dbionils@gmail.com (N.F.); pcoila@unap.edu.pe (P.C.); 4 Facultad de Medicina Veterinaria y Zootecnia, Universidad Nacional Micaela Bastidas de Apurímac, Abancay 03001, Peru; capetillo_45@hotmail.com; 5 Facultad de Ingeniería y Ciencias Agrarias, Universidad Nacional Toribio Rodríguez de Mendoza de Amazonas (UNTRM), Amazonas 01001, Peru; 6 Centro de Investigación en Germoplasma Vegetal y Mejoramiento Genético de Plantas (CIGEMP), Universidad Nacional Toribio Rodríguez de Mendoza de Amazonas (UNTRM), Amazonas 01001, Peru

**Keywords:** microRNAs, organ-specific gene expression, functional enrichment analysis, NGS

## Abstract

Background: MicroRNAs (miRNAs) play crucial roles in regulating tissue-specific gene expression and plant development. This study explores the identification and functional characterization of miRNAs in *Plukenetia volubilis* (sacha inchi), an economically and nutritionally significant crop native to the Amazon basin, across three organs: root, stem, and leaf. Methods: Small RNA libraries were sequenced on the Illumina Novaseq 6000 platform, yielding high-quality reads that facilitated the discovery of known and novel miRNAs using miRDeep-P. Results: A total of 277 miRNAs were identified, comprising 71 conserved and 206 novel miRNAs, across root, stem, and leaf tissues. In addition, differential expression analysis using DESeq2 identified distinct miRNAs exhibiting tissue-specific regulation. Notably, novel miRNAs like novel_1, novel_88, and novel_189 showed significant roles in processes such as auxin signaling, lignin biosynthesis, and stress response. Functional enrichment analysis of miRNA target genes revealed pathways related to hormonal regulation, structural reinforcement, and environmental adaptation, highlighting tissue-specific functions. The Principal Component Analysis and PERMANOVA confirmed clear segregation of miRNA expression profiles among tissues, underlining organ-specific regulation. Differential expression patterns emphasized unique regulatory roles in each organ: roots prioritized stress response and nutrient uptake, leaves focused on photosynthesis and UV protection, and stems contributed to structural integrity and nutrient transport, suggesting evolutionary adaptations in *P. volubilis*. Conclusions: This study identified novel miRNA-mediated networks that regulate developmental and adaptive processes in *P. volubilis*, underscoring its molecular adaptations for resilience and productivity. By characterizing both conserved and novel miRNAs, the findings lay a foundation for genetic improvement and molecular breeding strategies aimed at enhancing agronomic traits, stress tolerance, and the production of bioactive compounds.

## 1. Introduction

*P. volubilis*, commonly known as sacha inchi, is a plant highly valued for its nutritional profile and health benefits. Native to the Amazon basin and now cultivated in countries like Peru and Thailand, this plant is notable for its seeds, which are rich in polyunsaturated fatty acids such as α-linolenic acid and linoleic acid, along with tocopherols that provide antioxidant and anti-inflammatory properties [1]. These compounds make sacha inchi seeds an attractive source for the food, nutraceutical, and pharmaceutical industries. Additionally, other plant organs, such as leaves and shells, exhibit antioxidant activity and potential for heavy metal bioremediation, expanding the applications of this versatile crop [2].

Studying the organ development of *P. volubilis* is essential for understanding its nutritional and medicinal value and optimizing its cultivation. By examining the transcriptomic profiles of main organs, such as roots, stems, leaves, flowers, and seeds, researchers can identify specific gene expressions associated with the biosynthesis of bioactive compounds [2,3]. This knowledge supports genetic improvement programs and optimized cultivation practices to enhance the production of essential oils and nutrients [2].

MicroRNAs (miRNAs) in plants play key roles in regulating gene expression, impacting growth, development, and responses to stress. These small, non-coding RNAs, typically 20–24 nucleotides long, participate in post-transcriptional regulation by directing the degradation or inhibiting the translation of specific mRNAs [4]. Profiling miRNAs in plants offers valuable insights into these regulatory mechanisms, and studies in species like *Arabidopsis* have identified essential proteins such as ARGONAUTE (AGO) and Dicer-like (DCL) that process and guide miRNAs, enabling the precise regulation of gene expression [5,6].

Illumina sequencing of miRNAs has become a central method for analyzing small RNA transcriptomes due to its high throughput and precision [7]. This technology allows the simultaneous analysis of multiple samples by generating millions of reads from small RNA-derived cDNA libraries, although adapter bias and other technical factors can impact miRNA quantification. Despite these challenges, Illumina sequencing remains efficient for obtaining high-resolution miRNA profiles, offering insights into gene regulation across various biological contexts [7,8,9].

Transcriptomic studies in *P. volubilis* have provided critical information on gene expression and molecular responses across developmental stages and under stress conditions. A recent analysis of seeds at two developmental stages (Pv-1 and Pv-2), using RNA-Seq and iTRAQ, identified 53,224 unigenes and 6026 proteins, with 8815 differentially expressed genes (DEGs) and 4983 differentially abundant proteins (DAPs), respectively. This study highlights high expression levels of ribosome-inactivating protein (RIP) transcripts in Pv-2, suggesting bioactive potential for these compounds in oil safety and antiviral activity in roots [10]. Additional studies have identified lipid biosynthesis genes responsive to heat stress, such as DGAT2, which increases triacylglycerol (TAG) accumulation to mitigate cellular damage, contributing to high-quality oil production [11]. Moreover, de novo transcriptome assembly has revealed 124,750 unique transcripts across various organs, highlighting genes involved in α-linolenic acid (ALA) biosynthesis in seeds and other organ-specific expression patterns, laying a solid foundation for the genetic improvement of *P. volubilis* [1].

This study is based on the hypothesis that the microRNA (miRNA) profile in *P. volubilis* (sacha inchi) varies among specific organs—root, leaf, and stem—and that these miRNAs play key roles in regulating genes associated with the biosynthesis of bioactive compounds and plant development. The main objective was to characterize the miRNA profile in these three organs of Peruvian sacha inchi, identifying differentially expressed miRNAs and their target genes related to essential metabolic processes. In the future, the findings from this study could lay the groundwork for the genetic improvement of *P. volubilis*, focusing on optimizing its cultivation and harnessing its nutraceutical and pharmaceutical properties.

## 2. Materials and Methods

### 2.1. Cultivation of Sacha Inchi and Sample Preparation

The seeds were sterilized using 10% sodium hypochlorite for 15 min and subsequently washed multiple times with sterile water. Afterward, they were treated with 37% sulfuric acid for 5 min, rinsed with water to remove any residues, and soaked in water at room temperature for 3 days to promote hydration. Subsequently, plantlets were transplanted in substrate and cultivated in a growth chamber TPC-37 (BioChambers, Inc., Winnipeg, MB, Canada) at the Instituto Nacional de Innovación Agraria (INIA) under controlled conditions (22 °C/night at 18 °C with a photoperiod of 12 h light–1600 lux/12 h dark), and under a constant humidity of 80%. Roots, stems, and leaves were gathered from 3-month-old sacha inchi plants during the initial stage of flower bud development, a critical phase where key regulatory processes, such as organ differentiation and hormonal signaling, are activated. Sampling at this stage enables the identification of developmentally and tissue-specific miRNAs involved in pathways essential for growth, stress response, and adaptation [12]. Three distinct biological samples were prepared for each tissue type to ensure the robust representation of miRNA expression profiles.

### 2.2. Extraction of smallRNA and Sequencing of microRNA

MicroRNAs were isolated using the Norgen microRNA Purification Kit (Norgen Biotek Corp., Thorold, ON, Canada) following the manufacturer’s protocol. The concentration of the extracted miRNAs was quantified with the Qubit microRNA Assay Kit (Thermo Fisher, Waltham, MA, USA), and the integrity of the miRNAs was assessed using the Bioanalyzer Small RNA Analysis Kit (Agilent, Hilden, Germany). After confirming that the sample quality complied with the sequencing standards, miRNA libraries were constructed using the NEBNext Multiplex Small RNA Library Prep Set for Illumina (NEB, Ipswich, MA, USA), adhering to the manufacturer’s instructions. Subsequently, the indexed samples were grouped on the cBot Cluster Generation System with the TruSeq SR Cluster Kit v3-cBot-HS (Illumina, San Diego, CA, USA). Lastly, the library preparations underwent sequencing on the Illumina Novaseq 6000 platform with single-end 50 bp sequencing strategy. The associated Bioproject and Biosample numbers are PRJNA1192310, SAMN45105876–SAMN45105884.

### 2.3. Pre-Processing of Sequencing Data

The raw FASTQ files underwent quality control analyses using software FastQC v0.11.9 [13]. Adapter sequences were subsequently removed using program Trim Galore [14] with default parameters. Reads were filtered by length, retaining only those between 16 and 40 bp. Afterwards, sRNAs derived from rRNAs, scRNA, snRNAs, snoRNAs, tRNA, and repetitive elements (RE) from the SILVA rRNA [15], Rfam [16], RNAcental [17], tRNAdb [18], NCBI [19], and PlantRep [20] were identified by mapping the clean reads with Bowtie v 1.3.1 [21] without mismatches [22]. The remaining unaligned reads along with reads aligned to miRNA sequences were used for downstream analysis.

### 2.4. Identification of Known and Novel microRNAs

The miRDeep-P software v1.3 [23] was employed to predict mature, star, and precursor sequences from the transcriptome of *P. volubilis*. Given the absence of a reference genome for *P. volubilis*, the transcriptome (Bioproject: PRJNA377968) was used for this purpose. The core module of miRDeep-P used the *P. volubilis* transcriptome along with known precursors and mature miRNAs from *Manihot esculenta* to perform these predictions, as *M. esculenta* was the closest relative to *P. volubilis* available in the miRBase database (Release 22.1, October 2024) [24,25]. In addition, the quantifier module of miRDeep-P was used to quantify the expression levels of conserved miRNAs. This module aligned sequencing reads to known conserved miRNA precursors from miRBase, calculating read counts for the corresponding mature miRNAs. The secondary structures of the precursor miRNAs and detailed alignment reports were generated for each predicted miRNA from both modules.

The prediction of secondary structures, a key step in miRNA identification, was performed using RNAfold from the ViennaRNA v2.6 package [26], integrated into miRDeep-P. RNAfold calculates the minimum free energy (MFE) of RNA sequences, with lower MFE values indicating greater structural stability [27,28], a hallmark of biologically plausible precursor miRNAs [29,30]. miRDeep-P incorporates MFE values into its scoring system, assigning higher confidence to candidates with stable stem-loop configurations and low MFEs, thereby enhancing the biological relevance of its predictions [23].

The identified miRNAs were classified into families based on their sequence similarity to reference miRNAs from the miRBase database. This classification was derived from the final reports generated by the core and quantifier modules of miRDeep-P, which indicate the closest matching reference miRNA for each identified sequence. miRNAs with fewer than two mismatches relative to the reference miRNA were classified as belonging to the same family.

### 2.5. Differential Expression Analysis of miRNAs Between Organs

The differential expression of miRNAs was analyzed using DESeq2 [31], starting with raw counts from miRDeep-P. To handle zero counts, a constant of 1 was added to all raw counts, which were then rounded. The modified data, along with sample-specific metadata, were used as input. The experimental design incorporated the different tissue types—root, stem, and leaf—as a condition in the design formula. miRNAs with low overall counts (those with a sum of counts across samples ≤ 5) were filtered out to reduce the influence of noise on the analysis, thus retaining only miRNAs with sufficient overall expression levels for meaningful analysis. Normalization of the raw counts and subsequent differential expression analysis were performed using DESeq2’s core function, DESeq. DESeq2 uses size factors to correct for variations in library size across samples. The size factor for each sample (*Size Factor*_j_) was determined using the median ratio method. In this approach, the geometric mean of counts for each miRNA across all samples was first calculated:

$$Geometric\;Mean_{i}=(\prod_{j=1}^{n}\;Raw\;Count_{ij})$$

where *n* is the number of samples and Raw Count_*ij*_ is the count for miRNA_i_ in sample_j_. Then, for each sample, a size factor was estimated by computing the median of the ratios between the raw counts for each miRNA and the geometric mean across samples:

$$Size\;Factor_{j\;\;\;}=median(\frac{Raw\;Count_{ij}}{Geometric\;Mean_{i}})$$

Finally, the normalized count for each miRNA_i_ in sample_j_ was subsequently computed as follows:

$$Normalized\;Count_{ij}=\frac{Raw\;Count_{ij}}{Size\;Factor_{j\;\;\;}}\;$$

These normalized counts were used for differential expression analysis, thereby ensuring reliable assessments of miRNA differential expression across organ tissues. The analysis included three pairwise comparisons between tissue types: (1) root vs. leaf, where miRNA levels in root tissue were compared to those in leaf tissue; (2) leaf vs. stem, where expression was compared between leaf and stem tissues; and (3) stem vs. root, comparing the stem to root tissues. The differential expression analysis for each comparison was performed using DESeq2’s results () function, with specific contrasts used to extract the desired pairwise comparison results. In each comparison, the first condition served as the numerator for the log2 fold change (log2FC) calculation, and the second condition served as the denominator. This ensured a consistent interpretation of the log2FC values, indicating whether the expression was higher or lower in the reference condition.

Following differential expression analysis, miRNAs were filtered to identify significantly differentially expressed candidates. Only miRNAs meeting the criteria of an adjusted *p*-value (false discovery rate, FDR) below 0.05 and an absolute log2 fold change greater than 1 were selected as significantly differentially expressed. This filtering approach ensured that only biologically meaningful changes in expression were considered for subsequent analyses. Significant miRNAs identified through this process were further visualized using a combination of R v4.2.1 packages: volcano plots were created with EnhancedVolcano v1.6.0 [32], Venn diagrams were generated with ggVennDiagram v1.2.2, and expression patterns were depicted through heatmaps using Pheatmap v1.0.12.

### 2.6. Multivariate Analysis of miRNA Expression Profiles

The normalized counts were extracted from the DESeq2 result and used to generate principal component analysis (PCA) plots to visualize the variance and clustering of miRNA expression profiles among different organ tissues (leaf, root, stem). Two sets of PCA plots were generated: one including both novel and known (conserved) miRNAs and a second including only the novel miRNAs identified in the tissues. These two approaches were intended to evaluate the extent to which novel miRNAs and the overall miRNA pool (including conserved miRNAs) could contribute to differences in tissue-specific expression profiles. PCA was employed to assess whether distinct patterns of miRNA expression could explain the separation between different tissue types. To further quantify these differences, PERMANOVA (Permutational Multivariate Analysis of Variance) was conducted for both PCA analyses, using the adonis2 function from the vegan R package [33], testing for significant differences in miRNA expression profiles in the multivariate space defined by different tissues. This analysis was performed to determine if there were significant clustering patterns among the samples based on their tissue of origin. The plots were generated using the ggplot2 v3.3.3 R package [34].

### 2.7. Prediction of Target Genes

The prediction of putative target genes regulated by the miRNAs identified in our samples was conducted using the psRNATarget web server [35]. For this analysis, we used the transcript dataset of *Arabidopsis thaliana* from the JGI genomic project (Phytozome 13, 447_Araport11). This dataset is derived from the Araport11 annotation, which represents the latest and most comprehensive annotation of transcribed sequences, built upon the TAIR10 genome assembly. The target prediction scoring scheme employed was based on version 2 of the psRNATarget scoring system, released in 2017. The prediction parameters included a maximum expectation value of 5, a complementarity scoring length (HSP size) of 19 nucleotides, and a maximum of two mismatches allowed in the seed region.

### 2.8. Functional Enrichment Analysis of Target Genes: GO and KEGG Pathways

Functional enrichment analysis of the target genes was conducted using Gene Ontology (GO) and KEGG pathway enrichment to identify overrepresented biological processes and pathways influenced by the target genes of differentially expressed miRNAs for each organ comparison (stem vs. root, leaf vs. stem, root vs. leaf) in *P. volubilis*. The target genes were classified based on the expression status of their corresponding miRNAs, resulting in two gene sets for each comparison: genes targeted by upregulated miRNAs and genes targeted by downregulated miRNAs.

For GO enrichment analysis, the clusterProfiler [36] and topGO [37] R packages were used. The compareCluster function from clusterProfiler applied the enrichGO function to identify enriched GO terms in the Biological Process ontology using the org.At.tair.db Arabidopsis database [38], with statistical significance defined by a *p*-value cutoff of 0.05 (adjusted using the Benjamini–Hochberg method, BH) and a q-value cutoff of 0.2. Additionally, topGO was used for complementary GO enrichment analysis, using Fisher’s exact test with the “classic” algorithm, excluding GO terms with fewer than 10 genes. Dot plots were generated to visualize and compare enriched GO terms for both upregulated and downregulated gene sets.

For KEGG pathway enrichment analysis, clusterProfiler was also used with enrichKEGG to analyze target genes identified by psRNATarget for pathway enrichment. The compareCluster function facilitated comparisons, specifying the Arabidopsis code “ath.” Enrichment significance was assessed using the same *p*-value and q-value criteria as for GO enrichment. Dot plots were generated to visualize the enriched pathways associated with upregulated and downregulated miRNA targets.

## 3. Results

For the identification of miRNAs in *P. volubilis*, a total of 99,512,787 raw reads were sequenced from nine small RNA libraries, with three libraries prepared for each organ: leaves, stems, and roots. These reads were then analyzed to remove adapters and low-quality reads. Additionally, reads that were not between the range of 16 and 40 bp were also removed, leaving 45,497,479 clean, high-quality reads.

Afterward, reads corresponding to other RNA types were identified (Table 1). Ribosomal RNAs (rRNAs) and transfer RNAs (tRNAs) were the most abundant, with rRNAs accounting for 10,233,777 reads in the three organs: 1,574,564 reads in leaves, 2,269,843 in stems, and 6,389,370 in roots. Reads mapping to repetitive elements (REs) were significant in leaves (2,227,046), but much lower in stems and roots. Non-coding RNAs, including snRNAs, snoRNAs, and scRNAs, had low representation but varied across tissues. Messenger RNAs (mRNAs) and miRNAs were moderately abundant, with miRNAs contributing 335,241 reads in leaves, 720,488 in stems, and 161,407 in roots. A large proportion of reads (54.8%) remained unaligned. Only these, along with reads aligned to miRNA sequences, were used for further analysis.

### 3.1. Profile of Known and Novel microRNAs

**Figure 1:**
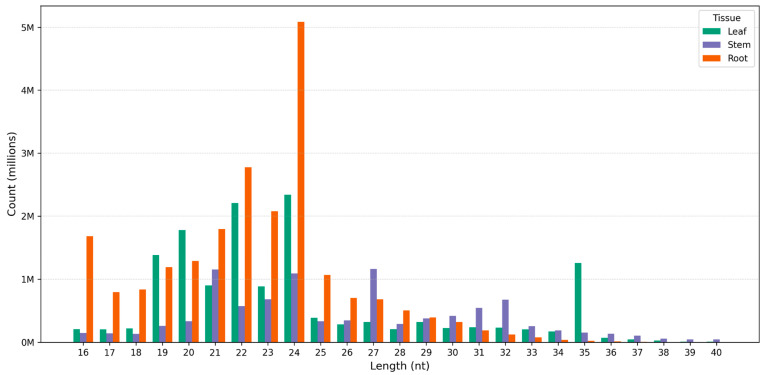
Distribution of clean reads with different sequence lengths according to their total read counts (in millions) in root (MR), stem (MS), and leaf (ML) tissues.

The distribution of clean reads by sequence length shows distinct patterns among the tissues (leaf, stem, and root) (Figure 1). The 24 nt reads are the most abundant in the root tissue, with a sharp peak surpassing other length. In contrast, the 21 nt reads are relatively more frequent in the stem and leaf tissues compared to the root. The third most frequent read length in the root is 23 nt, followed by 22 nt, whereas in the stem and leaf, the 20 nt reads show higher abundance. Some reads with low counts (<2 instances) across replicates were likely excluded as sequencing errors, while those sequenced over one hundred times were considered relatively highly expressed.

**Figure 2:**
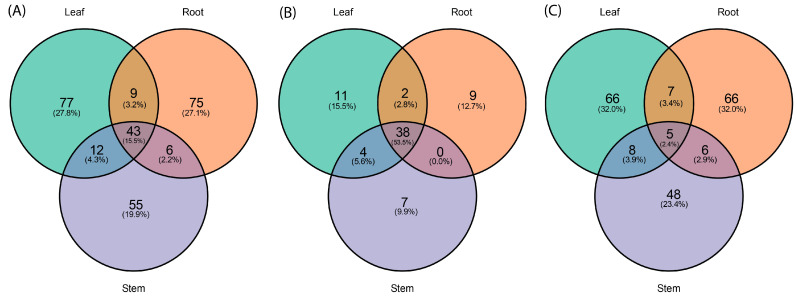
Venn diagram showing the shared and distinct microRNAs identified across the root, stem, and leaf small RNA libraries. The miRNAs shown range in size from 17 to 25 nt. (**A**) Distribution of all miRNAs; (**B**) only known miRNAs; and (**C**) only novel miRNAs.

A total of 277 miRNAs were identified across the three organs of *P. volubilis*, comprising 71 conserved miRNAs and 206 novel miRNAs. The distribution of known and novel miRNAs across the three organs is presented in Figure 2A. As shown, the three organs share 43 miRNAs, comprising 38 known miRNAs (Figure 2B) and 5 novel miRNAs (Figure 2C). In total, 77 miRNAs are unique to leaves, of which 11 are known (Figure 2B) and 66 are novel (Figure 2C); roots contain 75 unique miRNAs, including 9 known and 66 novel miRNAs (Figure 2B,C); stems include 55 unique miRNAs, with 7 known and 48 novel miRNAs (Figure 2B,C).

**Figure 3:**
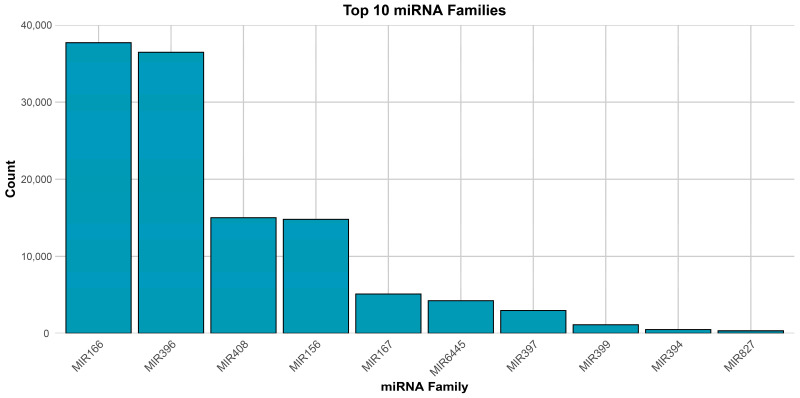
The top 10 miRNA families and their respective reads among three organs. The miR166 family has the highest number of respective reads among all of the miRNA families.

The bar chart presents the 10 most abundant miRNA families (Figure 3), with miR169 and miR396 exhibiting the highest counts, each exceeding 30,000 occurrences. miR396 is characterized by 51 family members, highlighting its significant presence across diverse samples. Similarly, miR169 consists of 18 family members, indicating its broad conservation and functional relevance. In particular, miR166 and miR158 maintain counts above 10,000, with miR166 having 52 family members. miR160, although not as highly expressed, consists of 45 family members, emphasizing its conservation in regulating vital pathways. Other miRNAs, such as miR167, miR164, miR482, miR159, miR394, and miR827, demonstrate progressively lower counts but remain functionally relevant in specific processes.

**Figure 4:**
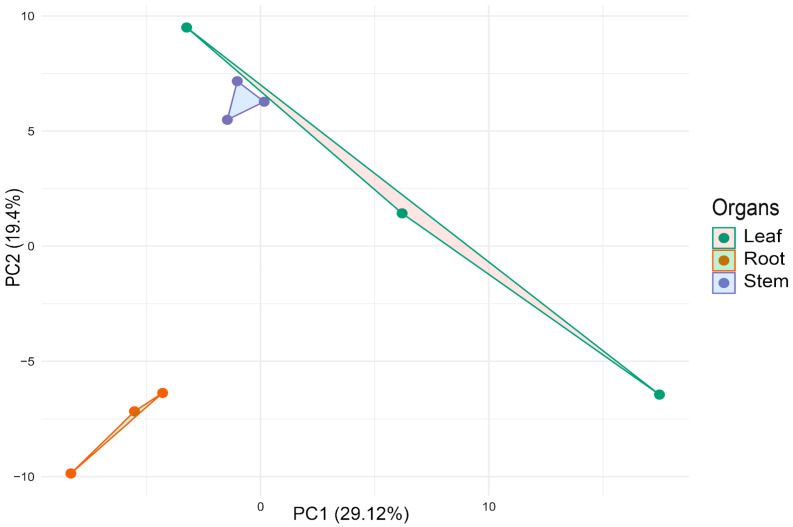
Principal Component Analysis (PCA) plot showing the unsupervised clustering based on total miRNA (novel and known) expression of all samples.

The principal component analysis based on Euclidean distance for sacha inchi samples derived from root, stem, and leaf tissues of both novel and known microRNAs revealed distinct separations between root and leaf, as well as root and stem samples, with a slight differentiation also observed between leaf and stem (Figure 4). This pattern was supported by PERMANOVA results, yielding a *p*-value of 0.013 (Table 1).

Additionally, a separate PCA using only novel microRNAs demonstrated clear segregation among sacha inchi organs (Figure S1), further confirmed by PERMANOVA analysis with a *p*-value of 0.0046 (Table 2).

### 3.2. Differential Expression Analysis of miRNAs and Their Target Genes

**Figure 5:**
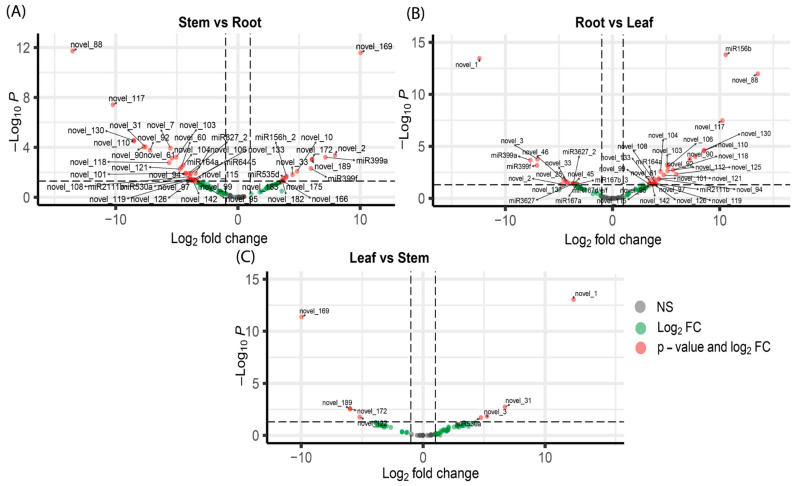
Volcano plots of differentially expressed miRNAs from pairwise comparisons of *P. volubilis* organ tissues. (**A**) Volcano plot of the stem vs. root comparison. (**B**) Volcano plot of the root vs. leaf comparison. (**C**) Volcano plot of the leaf vs. stem comparison.

Differential expression analysis of miRNAs across the three organ tissues revealed distinct regulatory patterns, as depicted in the volcano plots (Figure 5). In the stem vs. root comparison, 45 miRNAs exhibited significant differential expression (Figure 5A). Notably, novel_169 was highly upregulated in stem tissue, as indicated by a positive log2 fold change (log2FC), reflecting higher expression in the stem compared to the root. Conversely, novel_88 was significantly downregulated in the stem, with a negative log2FC, suggesting higher expression in the root.

For the root vs. leaf comparison (Figure 5B), 43 miRNAs were differentially expressed. For instance, novel_1 showed significant downregulation in the root, represented by a large negative log2FC, indicating higher expression in leaf tissue. On the other hand, miR156b and novel_88 were significantly upregulated in the root, with positive log2FC values, indicating higher expression in the root compared to the leaf.

In the leaf vs. stem comparison (Figure 5C), miRNAs such as novel_1 and novel_31 were significantly upregulated in the leaf tissue, as evidenced by positive log2FC values, indicating higher expression in the leaf compared to the stem. Conversely, novel_169 and novel_189 were downregulated in leaves, with negative log2FC values, indicating lower expression in the leaf tissue and higher expression in the stem tissue. Notably, the leaf vs. stem comparison had lower amounts of differentially expressed miRNAs compared to the other two comparisons, showing only eight miRNAs differentially expressed. These results collectively highlight that specific miRNAs are involved in the unique regulatory functions of each tissue, emphasizing their specialized roles in plant growth and development.

**Figure 6:**
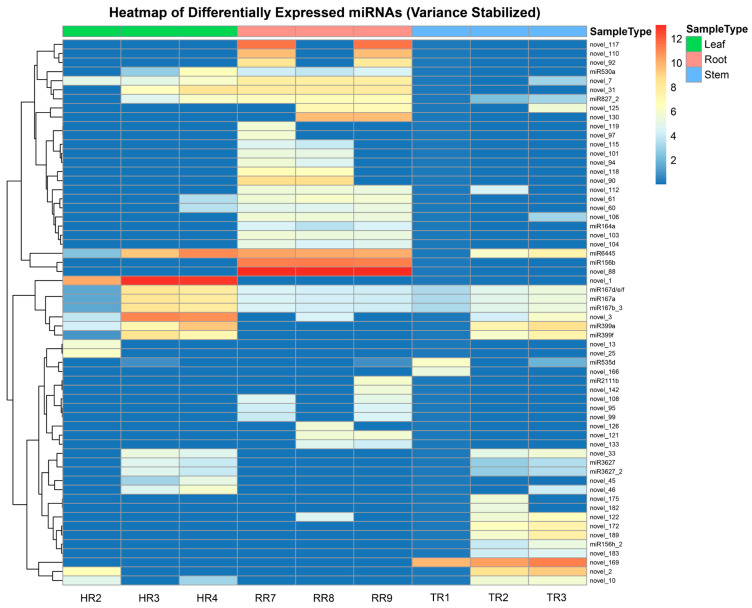
Heatmap displaying the differentially expressed miRNAs across root, stem, and leaf samples. The *x*-axis represents individual biological replicates for each organ: HR2, HR3, and HR4 correspond to leaf samples; RR7, RR8, and RR9 correspond to root samples; TR1, TR2, and TR3 correspond to stem samples. The *y*-axis lists the differentially expressed miRNAs.

A total of 60 miRNAs exhibited distinct differential expression patterns across the three tissue types: leaf, root, and stem. The heatmap and hierarchical clustering analysis demonstrated clear separations in the miRNA expression profiles among the different tissue types (Figure 6).

The analysis revealed two main clusters of miRNAs, each containing subclusters with unique expression patterns. The first cluster consisted of miRNAs with elevated expression in root samples, including novel_117, novel_88, miR156b, and miR6445, and these miRNAs exhibited lower expression in leaf and stem tissues. In contrast, the second cluster included miRNAs predominantly expressed in leaf samples, such as novel_1, miR167d/e/f, miR167a, miR167b_3, novel_3, miR399a, and miR399f, as well as miRNAs with higher expression in stem samples, including novel_169, novel_2, novel_10, and novel_189, with minimal expression in the root. These expression patterns indicate a high degree of tissue specificity among miRNAs, with each tissue displaying a distinct miRNA profile. Such differentiation in miRNA expression may reflect underlying molecular mechanisms tailored to the physiological requirements of each tissue type.

### 3.3. Potential Targets of Novel miRNAs in Sacha Inchi

The novel miRNAs identified in Plukenetia volubilis demonstrated significant regulatory potential, with their predicted target genes spanning diverse biological processes and pathways crucial for plant development, stress response, and environmental adaptation (Table S1). These miRNAs, uniquely identified as novel to *P. volubilis*, exhibited target genes predominantly associated with auxin metabolism, meristematic phase transitions, and transmembrane transport, indicating their role in maintaining organ-specific growth and differentiation (Table 3). In contrast, downregulated novel miRNAs were linked to targets involved in lignin biosynthesis, vacuolar transport, and defense responses, suggesting a de-repression of these processes in specific organs to support specialization and enhance stress resilience. The diversity of target genes, including those regulating hormone signaling (e.g., ABI5 and AFP4), structural integrity (e.g., CAD2 and CAD3), and immune responses (e.g., RPS5 and CBP60b), highlights the intricate regulatory networks modulated specifically by these novel miRNAs. These results emphasize the critical role of the novel miRNA-mediated regulation in orchestrating developmental and adaptive processes in Plukenetia volubilis, offering new insights into its molecular mechanisms of organ differentiation and stress management.

**Figure 7:**
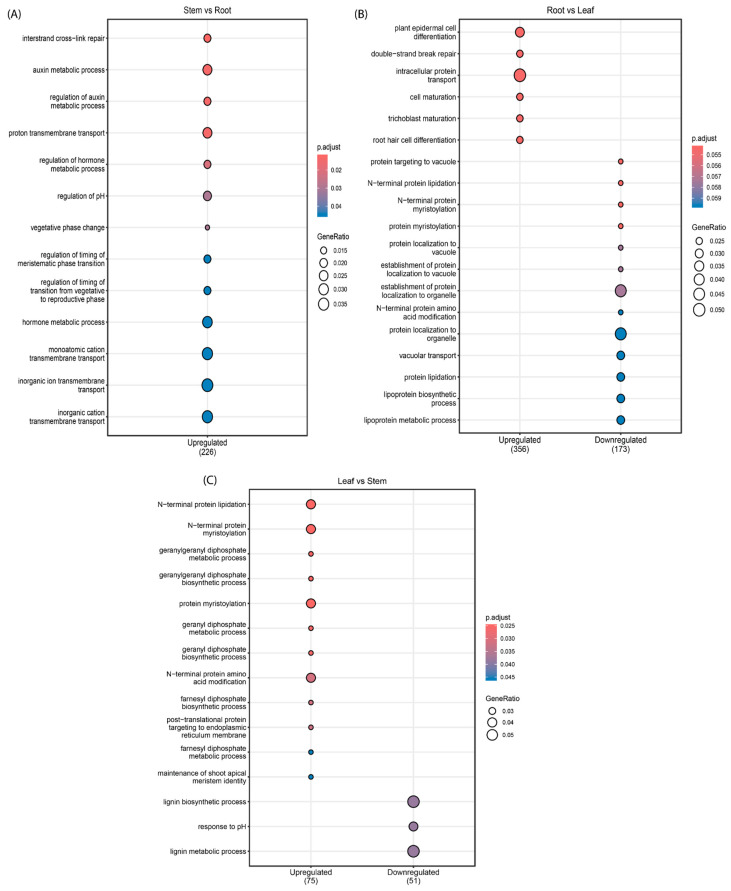
Dotplots displaying the results of the Functional enrichment (GO analysis) of the DE miRNA target genes in the three organ comparisons performed with ClusterProfiler. (**A**) GO enrichment analysis for DE miRNA target genes in stem vs. root (*p* < 0.05). (**B**) GO enrichment analysis for DE miRNA target genes in root vs. leaf (*p* < 0.06). (**C**) GO enrichment analysis for DE miRNA target genes in leaf vs. stem (*p* < 0.05). DE, differentially expressed; GO, gene ontology.

The results for the three comparisons revealed distinct regulatory patterns mediated by miRNAs (Figure 7). In the stem vs. root comparison, the target genes of the downregulated miRNAs were associated with several biological processes that are de-repressed in the stem and potentially repressed in the root tissues. In the stem, downregulated miRNAs were associated with the de-repression of processes related to growth and development, such as anatomical structure morphogenesis (GO:0009653), tissue development (GO:0009888), meristem development (GO:0048507), cell division (GO:0051301), and plant epidermal cell differentiation (GO:0090627). Processes related to cellular organization and transport, like establishment of localization in cells and intracellular transport, (GO:0051649, GO:0046907) were also de-repressed in the stem (Figure S2). On the contrary, the target genes of the upregulated miRNAs were linked to repressed processes in the stem that could potentially be active in the root, such as those involved in hormone signaling and growth regulation, like auxin metabolism (GO:0009850, GO:0090354), regulation of hormone metabolic processes (GO:0032350), and vegetative phase change (GO:0010050). Processes associated with reproductive and developmental transitions, such as timing of meristematic phase transitions (GO:0048506) and transition from vegetative to reproductive phases (GO:0048510), as well as flower development (GO:0009908), were also repressed in the stem. Additionally, other processes related to environmental and chemical homeostasis were also suppressed in the stem relative to the root, such as pH regulation (GO:0006885) and transmembrane transport of ions and protons (GO:0098655, GO:0098660, GO:0098662, GO:1902600) (Figure 7A).

In the root vs. leaf comparison, there were also several biological processes that were de-repressed in the root and potentially repressed in the leaf tissues (Figure 7B). Among them, processes related to the transport of proteins to vacuoles and other organelles were enriched (GO:0006623, GO:0072665, GO:0072666, GO:0072594, GO:0033365, GO:0007034). Additionally, processes related to protein targeting and membrane association, like protein lipidation and myristoylation (GO:0006498, GO:0006499, GO:0018377), also seemed more active in the root than in the leaf. Notably, processes linked to the defense and response to stimuli (GO:0006952, GO:0043207) and to lipoprotein biosynthesis and metabolism (GO:0042158, GO:0042157) were also de-repressed in the root. In contrast, biological processes associated with cell differentiation and development, such as plant epidermal cell differentiation (GO:0090627), root hair cell differentiation (GO:0048765), trichoblast maturation (GO:0048764), and cell maturation (GO:0048469), were found to be repressed in the root. Furthermore, processes like double-strand break repair (GO:0006302), as well as processes linked to protein transport and localization (GO:0015031, GO:0045184, GO:0046907), seemed to also be repressed in the root relative to the leaf.

For the leaf vs. stem comparison, the biological processes that were de-repressed in the leaf and potentially repressed in the stem tissues (Figure 7C) included those related to structural reinforcement and chemical defenses of plants in response to environmental stresses, such as lignin biosynthesis and phenylpropanoid metabolism (GO:0009809, GO:0009808, GO:0009699, GO:0009698). Processes connected to hormonal adjustments and lipid remodeling, like hormone (GO:0042445) and glycolipid metabolism, (GO:0006664) were also de-repressed. Additionally, processes associated with intracellular transport and response to stress, such as vesicle organization (GO:0016050), vesicle budding from membrane (GO:0006900), and cellular response to chemical stress (GO:0062197), were also found to be more active in the leaf relative to the stem. On the other hand, it was found that repressed processes due to upregulated miRNAs in the leaf (but potentially active in the stem) were related to protein targeting and membrane association, like N-terminal protein lipidation (GO:0006498) and protein myristoylation (GO:0018377).

Processes linked to secondary metabolites, particularly terpenoid biosynthesis (including Geranyl, Geranylgeranyl, and Farnesyl diphosphate biosynthesis; GO:0033384, GO:0033386, GO:0045337), were found to be repressed in the leaf tissues in relation to the stem. Notably, the maintenance of the shoot apical meristem identity (GO:0010492) was also repressed in the leaf, implying reduced meristematic activity in this tissue in comparison to the stem. Additionally, the analysis with topGO revealed defense-related processes repressed in leaves that could be de-repressed in the stem (Figure S2c), such as the response to biotic stimulus (GO:0009607), the response to external biotic stimulus (GO:0043207), the defense response to other organisms (GO:0098542), biological processes involved in interspecies interaction between organisms (GO:0044419), and the defense response (GO:0006952).

## 4. Discussion

The PCA analysis of miRNA expression profiles in *P. volubilis* revealed a clear separation between root, stem, and leaf tissues, emphasizing the tissue-specific regulation of miRNAs. This finding aligns with observations in other plants, where miRNAs show distinct expression patterns across organs to regulate gene expression and development. For example, in Arabidopsis thaliana, specific miRNAs are predominantly expressed in certain tissues, influencing organ development and function [39]. Similarly, in *Oryza sativa* (rice), miRNAs exhibit unique expression patterns that regulate genes related to nutrient uptake and hormonal pathways [40]. The pronounced differentiation between roots and other tissues in *P. volubilis* suggests that both known and novel miRNAs fulfill specialized roles suited to each organ’s functions. Additionally, the clear segregation in the PCA focusing on novel miRNAs highlights the critical role of uncharacterized regulatory elements in tissue-specific expression [41].

The differential expression of miRNAs, such as novel_88, novel_1, and novel_189, highlights their essential roles in regulating tissue-specific physiological processes, underscoring the complexity of miRNA-mediated gene regulation in plant growth and adaptation. Novel_88, predominantly upregulated in roots, targets genes like PIP1;3/PIP1C, KNAT6, and HSFA3. PIP1;3/PIP1C, an aquaporin, enhances membrane permeability, facilitating water transport, nutrient uptake, and osmotic stress response, which are vital for photosynthesis and biomass production under optimal conditions [42]. KNAT6, a KNOX transcription factor, regulates shoot apical meristem formation and organ boundary maintenance [43]. Similarly, HSFA3 plays a role in abiotic stress tolerance, particularly in the heat stress response through proline metabolism, enhancing thermotolerance while increasing sensitivity to salt stress [44]. The consistent upregulation of novel_88 in roots suggests its role in prioritizing water transport, osmotic adaptation, and pathogen defense while suppressing shoot-specific processes, aligning with root-specific functional requirements.

In contrast, novel_1 and novel_189 exhibit tissue-specific regulatory roles in leaves and stems, respectively. Novel_1, significantly upregulated in leaves, targets PIP2;5 and AtUVR8. PIP2;5 optimizes water and nutrient transport for photosynthesis and cellular turgor under variable humidity conditions [45]. AtUVR8, a UV-B photoreceptor, mediates photomorphogenic responses, reducing UV damage [46]. Novel_189, predominantly expressed in stems, targets LBD2, ATTPS7, and ATRAB-A2A, which are associated with lignin biosynthesis, nutrient transport, and cellular organization. LBD2 contributes to secondary cell wall formation and lignin deposition, essential for structural integrity [47]. ATTPS7 enhances stress resilience through trehalose metabolism [48], and ATRAB-A2A supports intracellular vesicle trafficking for cellular transport [49]. These distinct expression patterns demonstrate the specialized roles of novel_1 in promoting photosynthesis and UV protection in leaves, and novel_189 in supporting structural and metabolic functions in stems. Together, these findings highlight the pivotal role of miRNAs in coordinating tissue-specific gene networks, facilitating plant development and adaptation to environmental challenges.

The microRNAs identified in *P. volubilis* exhibited tissue-specific regulation, emphasizing their central role in the functional adaptation of each tissue. In roots, the high expression of miR156b and miR6445 suggests their involvement in regulating genes related to root architecture, nutrient uptake, and antioxidant responses, all critical for coping with abiotic stress in nutrient-poor soils [50,51,52]. MiR156b, through its regulation of SPL genes, influences root development and the accumulation of secondary metabolites, such as anthocyanins. Meanwhile, miR6445 enhances oxidative stress tolerance by modulating the antioxidant response via the AsA-GSH cycle [53].

In leaves, miR167d/e/f and miR399a/f play essential roles in auxin-mediated pathways and phosphate homeostasis, respectively. MiR167a regulates transcription factors like ARF6 and ARF8, which are crucial for reproductive organ development and osmotic stress resistance [54,55]. MiR399a and miR399f optimize phosphate translocation to aerial tissues by repressing the PHO2 gene, a key mechanism for photosynthesis and adaptation to tropical soils with high salinity or low fertility [56,57]. These miRNAs’ expression in leaves highlights their importance in metabolic regulation and resilience to environmental stress [58,59].

In stems, novel_169, novel_2, and novel_10 are predominantly expressed and may regulate lignification and vascular development, similar to miRNAs, such as miR857 and miR166/165 in Arabidopsis thaliana. MiR857 modulates laccases involved in lignin biosynthesis, while miR166/165 is associated with vascular cell differentiation [60,61]. The expression of these novel miRNAs in stems suggests their potential role in enhancing structural stability and nutrient transport in *P. volubilis*. Further studies are needed to confirm these functions.

The Gene Ontology (GO) enrichment analysis highlights a complex regulatory network governing tissue-specific development. In the stem vs. root comparison, several known miRNAs, including miR530a, miR827_2, miR156b, miR164a, miR2111b, and miR6445, were downregulated in stems compared to roots. The downregulation of miR2111b and miR827_2, which regulate phosphate homeostasis and nutrient transport under stress [62,63,64], may contribute to the de-repression of transport-related processes in stems. Similarly, the downregulation of miR156b and miR164a, known regulators of growth and development through SPL and NAC transcription factors [65,66], is associated with the activation of target genes involved in tissue development (GO:0009888), cell division (GO:0051301), and anatomical structure morphogenesis (GO:0009653) [67,68,69]. These findings suggest miR164a, a key regulator of lateral organ development via CUC genes [70,71], plays an important role in stem-specific growth processes. On the other hand, miRNAs upregulated in stems, such as miR399a, miR399f, miR156h, and miR535d, along with novel miRNAs, indicate complex regulatory strategies. miR156h, for instance, represses vegetative phase transitions (GO:0010050) and meristematic phase changes (GO:0048506) by targeting SPL genes [72,73]. miR399a/f, primarily associated with root function, appears to suppress ion transport and pH regulation (GO:0098655, GO:0006885) in stems [74,75]. The repression of auxin metabolism and hormone signaling pathways (GO:0009850, GO:0090354) in stems, despite no differential expression of classical auxin-related miRNAs (miR160, miR167, miR390) [76], hints at the involvement of novel miRNAs in regulating hormone pathways unique to *P. volubilis*. Especially notable are the opposing roles of miR6445, miR156h, and miR535d. The downregulation of miR6445 in stems, which confers drought tolerance via the miR6445-NAC029-GSTU23 module [77], aligns with the primary water-absorbing function of roots. In contrast, the upregulation of miR156h and miR535d, which promote stress tolerance and growth [78,79,80], suggests that stems adopt distinct stress response strategies. Together, these findings indicate that tissue-specific miRNA regulation in *P. volubilis* balances root-associated and stem-specific processes, supporting growth, stress adaptation, and nutrient homeostasis through coordinated regulatory mechanisms.

In the root vs. leaf comparison, the downregulation of specific miRNAs in roots appears to de-repress biological processes that support root-specific functions. The enrichment of defense-related terms, such as ‘defense response’ (GO:0006952) and ‘defense response to other organisms’ (GO:0098542), may be linked to the downregulation of miR167 family members. The reduced expression of miR167d is particularly significant, as studies in rice show its suppression enhances plant immunity against pathogens, suggesting a prepared state for biotic stress responses in root tissue [81]. Similarly, the downregulation of miR167a/b, which regulate auxin response factors (ARF6 and ARF8), could contribute to the de-repression of protein modification processes like ‘protein myristoylation’ (GO:0018377) and ‘protein targeting to vacuole’ (GO:0006623). These processes are essential for protein localization, signal transduction, and turnover, all critical for root development and environmental responses [82]. Additionally, the reduced expression of miR3627, involved in secondary metabolism and lignin biosynthesis [83], may enhance cell wall modifications and strengthen defense responses through its targets, WD40 proteins. These proteins support lignin biosynthesis and the production of defense-related compounds, aligning with roots’ role in structural support and pathogen defense [84]. This coordinated de-repression of defense and protein modification processes highlights roots as primary sites for stress responses and complex protein trafficking. On the other hand, the upregulation of miR156b, miR164a, and miR2111b in roots suggests regulation of developmental and cellular processes through target gene suppression in roots and de-repression in leaves. For instance, miR164a, which regulates organ development via NAC transcription factors, shows higher expression in roots, suppressing epidermal differentiation processes in leaves to allow the proper expression of NAC targets for leaf development [85]. The upregulation of miR2111b, which regulates root architecture as a mobile signal, is linked to enriched terms like ‘root hair cell differentiation’ (GO:0048765), emphasizing spatial control of root hair development [86]. The enrichment of ‘double-strand break repair’ (GO:0006302) may reflect miR156b’s role in developmental phase transitions, while the presence of novel miRNAs in roots suggests species-specific regulatory mechanisms [87]. Taken together, these findings indicate that conserved and novel miRNAs coordinate root-specific growth, development, and stress responses while maintaining distinct developmental programs in leaves.

In the leaf vs. stem comparison, the downregulation of novel miRNAs in leaves suggests the involvement of species-specific regulatory mechanisms in key biological processes. Processes related to lignin biosynthesis are particularly significant, as lignin is essential for structural support and defense in plants [88]. Novel_189 targets two critical enzymes—CCoAOMT1 and CAD2—key to monolignol biosynthesis, the building blocks of lignin [89,90,91,92]. Additionally, Novel_189 targets STOP1, a transcription factor regulating pH homeostasis under acidic stress, contributing to terms like cellular response to chemical stress (GO:0062197) and response to pH (GO:0009268) [93]. Novel_172 targets APX6, an enzyme detoxifying hydrogen peroxide, underscoring its role in oxidative stress response [94]. The enrichment of the hormone metabolic processes (GO:0042445) is linked to Novel_122 targeting ARF2 and DWF4 (CYP90B1). ARF2 represses auxin signaling and interacts with ethylene and ABA pathways, influencing development, while DWF4 is critical for brassinosteroid biosynthesis, a pathway supporting growth and development [95]. Novel_189 also targets ILL3, an enzyme releasing stored auxin, highlighting its role in hormonal regulation [96]. Inversely, the upregulation of miR530a and three novel miRNAs (Novel_1, Novel_3, and Novel_31) in leaves indicates tissue-specific defense regulation. miR530a, a negative regulator of plant immunity, modulates defense responses and may contribute to systemic acquired resistance [97,98]. Novel_3 targets genes like RCY1, ADR1-L1, and SOC3, which enhance immune responses [99,100,101,102,103].

The enrichment of shoot apical meristem maintenance processes is noteworthy, as these are typically regulated by well-known miRNAs, like miR166 and miR165 [104]. In *P. volubilis*, Novel_1 targets HB33 and ZHD5, transcription factors involved in ABA and auxin signaling during drought stress, influencing meristem development [105]. Novel_3 targets GGPPS3 and GGPPS4, enzymes responsible for producing geranylgeranyl diphosphate (GGPP), a precursor for isoprenoids involved in metabolism and development [106]. These findings in the leaf and stem comparison suggest that novel miRNAs in *P. volubilis* complement conserved regulatory mechanisms, enabling precise, tissue-specific control of development, stress adaptation, and metabolic processes.

The findings across all tissue comparisons suggest the potential importance of species-specific miRNAs in regulating a range of biological processes in *P. volubilis*. While their tissue-specific functions appear to involve roles in lignin biosynthesis, hormonal signaling, and stress responses, novel miRNAs may also contribute to maintaining specialized physiological roles in roots, stems, and leaves. This could indicate the adaptive potential of *P. volubilis*, where conserved and novel regulatory pathways might converge to fine-tune tissue-specific growth and environmental responses. These observations offer insights into miRNA-mediated regulation and could broaden our understanding of the molecular mechanisms underlying plant development and adaptation, with possible applications for improving crop resilience and productivity.

## 5. Conclusions

Our study reveals key insights into the miRNA landscape of *P. volubilis* (sacha inchi), an economically and nutritionally important plant with limited molecular characterization, and highlights the intricate interplay between conserved and novel regulatory pathways that control tissue-specific functions. By identifying conserved and novel miRNAs, we uncover regulatory networks involved in growth, stress responses, and secondary metabolism. Novel miRNAs, which target genes critical for lignin biosynthesis, hormonal signaling, and stress adaptation, highlight unique regulatory mechanisms in sacha inchi. These findings provide a foundation for advancing molecular breeding and biotechnological strategies to enhance the agronomic traits and stress resilience of this valuable crop.

## Figures and Tables

**Table 1:** RNA types in the small RNA libraries of *P. volubilis* leaf, stem, and root.

Read Data and Small RNA	Leaves	Stems	Roots
Raw reads	37,535,006	36,751,657	25,226,124
Clean, high-quality reads (16–40 bp)	14,148,212	9,669,204	21,680,063
rRNA	1,574,564	2,269,843	6,389,370
tRNA	1,668,102	637,753	1,264,217
Repeated Elements	2,227,046	316,027	1,034,962
snRNA	9898	14,532	23,789
snoRNA	24,241	16,797	33,475
scRNA	25,396	28,810	446
lncRNA	50	14	103
mRNA	660,540	306,365	809,679
miRNA	335,241	720,488	16,1407
Unaligned reads	7,623,134	5,358,575	11,962,615

**Table 2:** PERMANOVA analysis: Pr (>F) indicates significance at values less than 0.05.

	Df	R2	F	Pr (>F)
Organs	2	0.3375	1.528	0.013
Residual	6	0.6625		

**Table 3:** Subset of differentially expressed miRNAs and their predicted targets.

miRNA	Target Genes and Functions
Novel_122 (Down) Leaf vs. Stem	-ARF2: Auxin Response Factor
	-DWF4 (CYP90B1): Enzyme involved in the biosynthesis of brassinosteroids, a class of plant hormones derived from terpenoids.
Novel_172 (Down) Leaf vs. Stem	-CCS: Copper Chaperone for Superoxide Dismutase (CCS) -RBGD2: RNA-binding glycine-rich protein -NHX4: Sodium/hydrogen exchanger protein -GH9A4: Member of the glycoside hydrolase family 9 (GH9) -AGL52: MADS-box transcription factor -A: PX6Ascorbate peroxidase isoform
Novel_189 (Down) Leaf vs. Stem	-LBD2: LATERAL ORGAN BOUNDARIES DOMAIN transcription factor -NDR1/HIN1-like: protein associated with plant defense responses, upregulated during pathogen attack. -SNM1: Involved in DNA repair of interstrand cross-links -CCoAOMT1: Involved in lignin biosynthesis -DXPS3/DXS: A key enzyme for isoprenoid biosynthesis -ILL3: Involved in auxin metabolism, regulating hormone homeostasis.
Novel_1 (Up) Leaf vs. Stem	-NPF5.5: Nitrate transporter, which is interconnected with isoprenoid biosynthesis pathways. -HB33/ZHD5: Involved in regulating gene expression crucial for maintaining the identity and function of the shoot apical meristem.
Novel_3 (Up) Leaf vs. Stem	-GET3a and GET3c: Components of the Guided Entry of Tail-anchored proteins, which is involved in targeting tail-anchored proteins to the endoplasmic reticulum membrane. -GGPPS3/GGPS4: Enzymes that produce geranylgeranyl diphosphate (GGPP), a precursor for various isoprenoids, including chlorophylls, carotenoids, and gibberellins. -SOC3: Involved in regulating flowering time. Also involved with maintaining the identity of the shoot apical meristem (GO:0010492). -HRT/RCY1/RPP8: Involved in plant immunity mediating defense responses against pathogens. -PLDDELTA: Phospholipase D delta enzyme. Role in stress responses and membrane remodeling.
Novel_31 (Up) Leaf vs. Stem	WAK4: Wall-Associated Kinase 4, member of the wall-associated kinase family. Involved in cell elongation and response to pathogens. RPG2/SWEET13: Sugars Will Eventually Be Exported Transporter 13, a member of the SWEET family of sugar transporters. Responds to biotic stress by modulating sugar transport (availability).

## Data Availability

Bioproject PRJNA1192310.

## Supplementary Materials

The following supporting information can be downloaded at: https://www.mdpi.com/article/10.3390/genes16040417/s1, Figure S1: Principal Component Analysis (PCA) plot showing the unsupervised clustering based on total novel miRNA novel expression of all samples; Figure S2: Dotplots displaying the results of the functional enrichment (GO analysis) of the DE miRNA target genes in the three organ comparisons performed with topGO; Table S1: Differentially expressed novel miRNAs and their predicted targets.
