# Supplementary material for: Profiling of Known and Novel microRNAs in an Oleaginous Crop Native to the Amazon Basin, Sacha Inchi (Plukenetia volubilis), Through smallRNA-Seq
Source: Genes (Basel). 2025 Mar 31;16(4):417. doi: 10.3390/genes16040417 (PMC12026887; doi:10.3390/genes16040417)
Supplement: Supplementary file 1 [file genes-16-00417-s001.zip › Supplementary Figures.pdf]

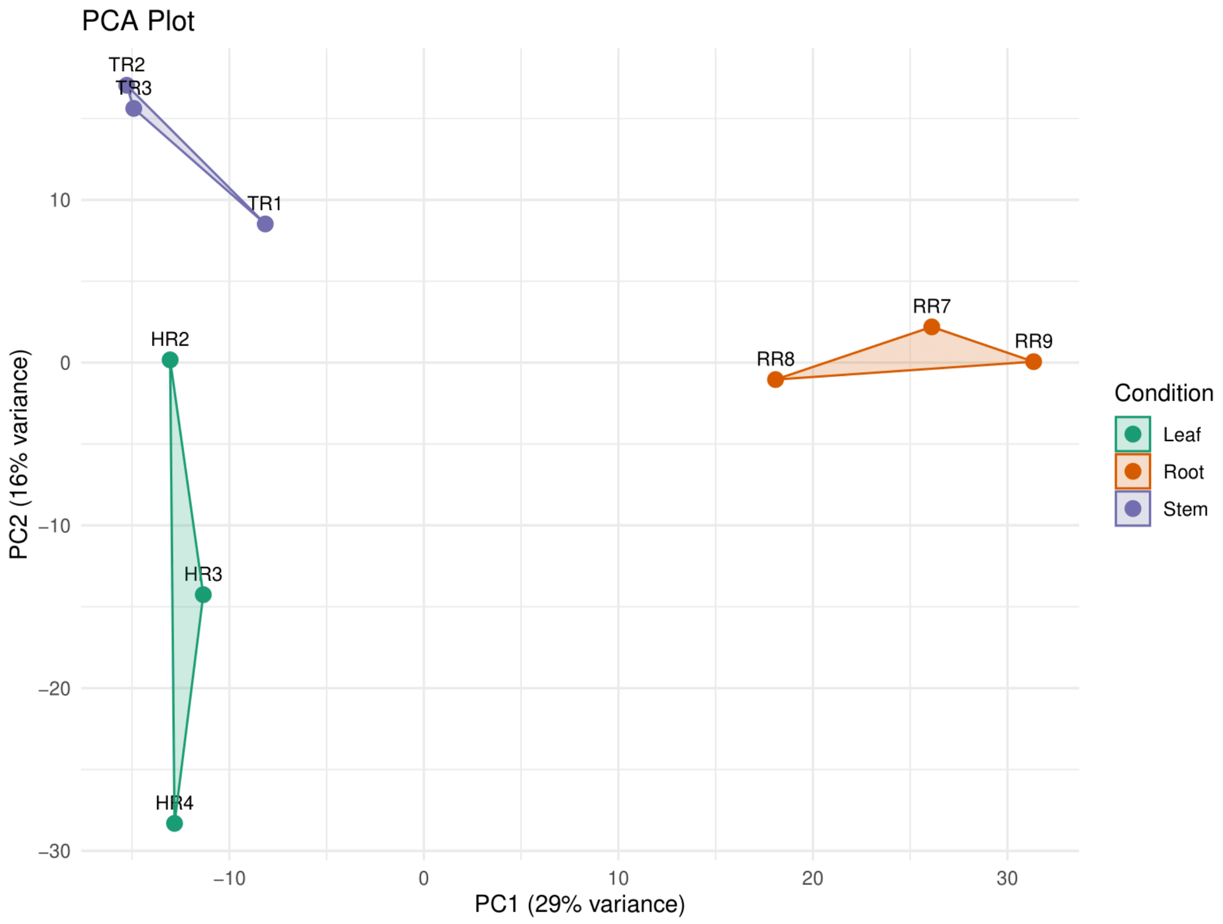

Figure S1. Principal Component Analysis (PCA) plot showing the unsupervised clustering based on total novel miRNA novel expression of all samples.

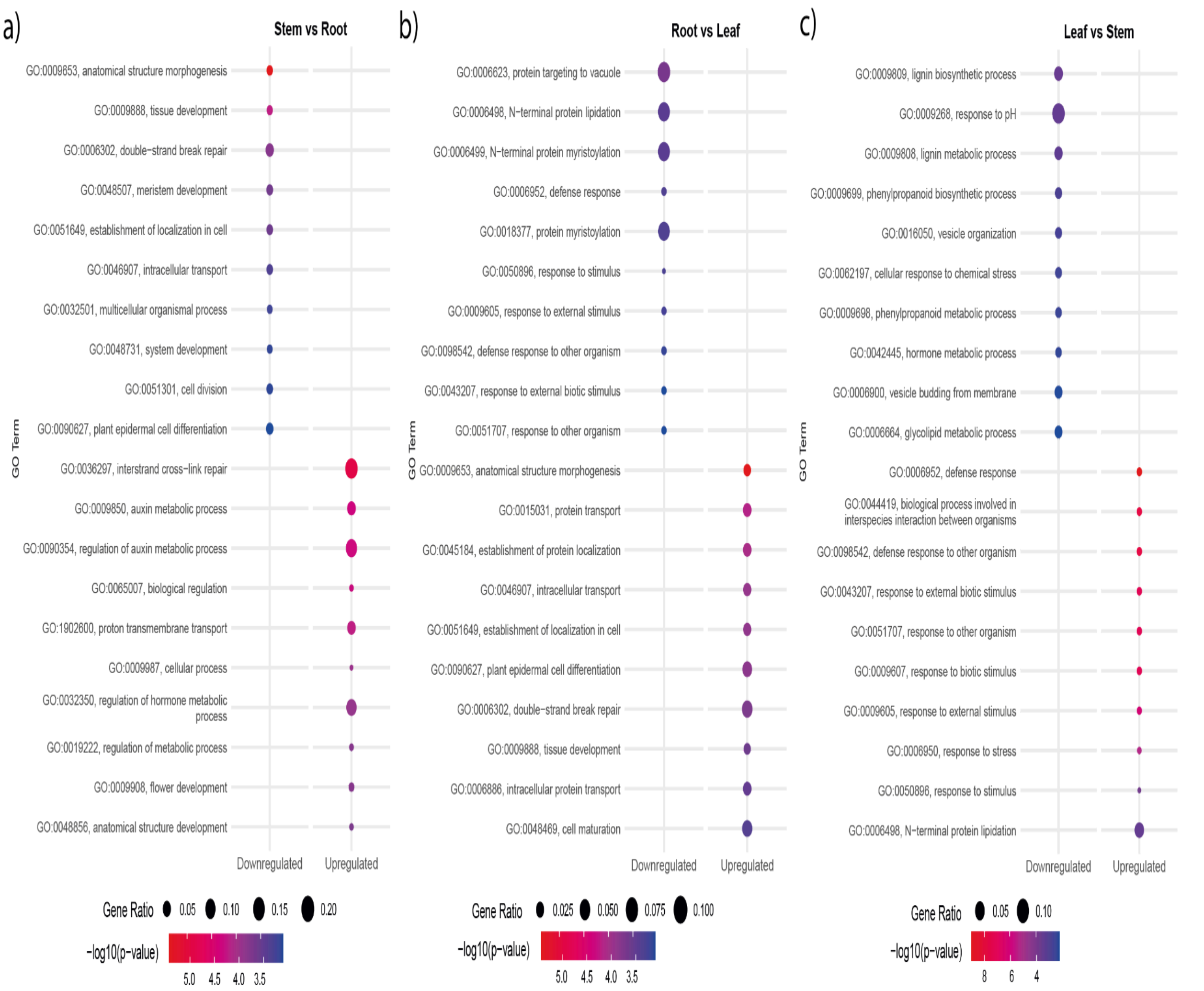

Figure S2. Dotplots displaying the results of the functional enrichment (GO analysis) of the DE miRNA target genes in the three organ comparisons done with topGO. (a) GO enrichment analysis for DE miRNA target genes in Stem vs. Root ($p < 0.05$). (b) GO enrichment analysis for DE miRNA target genes in Root vs. Leaf ($p < 0.05$). (c) GO enrichment analysis for DE miRNA target genes in Leaf vs. Stem ($p < 0.05$). DE, differentially expressed; GO, gene ontology
